# TRANSMISSION OF GENERALIZED TRUST AND BELIEF IN A JUST WORLD BETWEEN GRANDPARENTS AND ADULT GRANDCHILDREN

**DOI:** 10.1093/geroni/igae098.1601

**Published:** 2024-12-31

**Authors:** Tianyuan Li, Yue Yang Sun, Xiaoye Liu, Yukai Zhang

**Affiliations:** The Chinese University of Hong Kong (Shenzhen), Shenzhen, Guangdong, China (People’s Republic); The Chinese University of Hong Kong (Shenzhen), Shenzhen, Guangdong, China (People’s Republic); The Chinese University of Hong Kong (Shenzhen), Shenzhen, Guangdong, China (People’s Republic); The Chinese University of Hong Kong (Shenzhen), Shenzhen, Guangdong, China (People’s Republic)

## Abstract

Previous research shows that older adults tend to hold more positive beliefs about others and the social system compared to their younger counterparts. This study investigated whether such positive beliefs can be transmitted to the younger generation through intergenerational interactions. We recruited 292 Chinese adult grandchild-closest grandparent dyads and surveyed both members’ generalized trust and belief in a just world. Results revealed significant positive associations between grandparents’ and grandchildren’s generalized trust, β =.21, SE =.05, p <.001, and belief in a just world, β =.20, SE =.07, p =.004. While the intergenerational association of generalized trust was not moderated by other variables, that of belief in a just world was moderated by the grandchild’s familism, F(1, 279) = 4.46, p =.04, and parental intimacy, F(1, 279) = 7.94, p =.005. The intergenerational transmission of belief in a just world was not significant for the grandchildren with lower levels of familism, β =.02, p =.79, or parental intimacy, β =.02, p =.81; but was significant for those with higher levels of familism, β =.28, p =.003, or parental intimacy, β =.35, p <.001. Demographic variables were controlled in all the analyses. The findings underscore the importance of intergenerational transmission of positive social beliefs from grandparents to grandchildren, suggesting that older adults may play an essential role in cultivating social capital.

